# Influence of the timing of administration of crystalloid on maternal hypotension during spinal anesthesia for cesarean delivery: preload versus coload

**DOI:** 10.1186/1471-2253-14-36

**Published:** 2014-05-16

**Authors:** Ah-Young Oh, Jung-Won Hwang, In-Ae Song, Mi-Hyun Kim, Jung-Hee Ryu, Hee-Pyoung Park, Yeong-Tae Jeon, Sang-Hwan Do

**Affiliations:** 1 Department of Anesthesiology and Pain Medicine, Seoul National University College of Medicine, Seoul, South Korea; 2 Department of Anesthesiology and Pain Medicine, Seoul National University Bundang Hospital, Seongnam, South Korea; 3 Department of Anesthesiology and Pain Medicine, St. Mary’s Hospital, Catholic University of Korea School of Medicine, Seoul, Korea

**Keywords:** Cesarean delivery, Hypotension, Spinal anesthesia, Crystalloid

## Abstract

**Background:**

Prophylactic fluid preloading before spinal anesthesia has been a routine procedure to prevent maternal hypotension during cesarean delivery. Unlike colloid, timing of infusion of crystalloid may be important because of its short stay in intravascular space. We hypothesized that crystalloid loading just after intrathecal injection compared to preload would be more effective in preventing maternal hypotension.

**Methods:**

In this prospective controlled study, sixty parturients were randomized to receive 15 ml/kg of crystalloid before (preload group) or after (coload group) intrathecal drug injection for spinal anesthesia. Hypotension was defined if systolic arterial pressure decreased below 80% of baseline and ephedrine was administered to treat hypotension. The incidence of hypotension and the total dose of ephedrine were checked. Blood pressure, heart rate and nausea before childbirth were assessed. Neonatal outcomes were evaluated with Apgar scores and umbilical blood gas analysis.

**Results:**

The incidence of hypotension was lower in the coload group compared to the preload group (53% *vs.* 83%, P = 0.026). The blood pressure showed the bigger drop during spinal anesthesia in the preload group (34 ± 13 *vs.* 25 ± 10 mmHg, P = 0.002) and smaller dose of ephedrine was required in the coload group (7.5 [0–30] *vs*. 15 [0–40] mg, P = 0.015). The incidence of nausea was also lower in the coload group (27% *vs.* 60%, P = 0.019). Neonatal outcome measures were comparable between two groups.

**Conclusions:**

In case of using crystalloids for cesarean delivery, coload is more effective than preload for the prevention of maternal hypotension after spinal anesthesia.

**Trial registration:**

Clinical Research Information Service KCT0000324 (Jan 12^th^, 2012)

## Background

Spinal anesthesia is frequently used for cesarean delivery because of its rapid onset, a dense neural block, little risk of local anesthetic toxicity and minimal transfer of drug to the fetus, as well as little risk of failure of block. However, a higher incidence of hypotension is one of disadvantages of this technique. Intravenous administration of fluids, avoidance of aortocaval compression, and vigilant monitoring of blood pressure at frequent intervals are listed measures to decrease the risk of hypotension to varying degrees [1] but none have been shown to be sufficient [2].

Maternal hypotension becomes intensified by a deficit of intravascular volume adding to sympathetic blockade during spinal anesthesia. Traditionally, pre-hydration of fluids was recommended for the prevention of hypotension after spinal anesthesia. However, the efficacy of preload has been questioned and there were studies to evaluate the preventive effect of preloading of fluid comparing coload, that is, hydration at the time of actual block during cesarean delivery. Previous reports [3-5] which used colloid fluid, showed no significant differences in the incidence of maternal hypotension or in the neonatal outcome between the two methods. In regard to crystalloid, its effect is still on debate. Some studies [6,7] even found that prehydration using crystalloids had poor efficacy for prevention of hypotension during cesarean delivery. Although recent studies have shown that colloids are more effective in prevention of hypotension than crystalloids [8,9], many institutions are still using crystalloids because of potential disadvantages of colloids, such as cost, allergy, and effects on coagulation. Crystalloids do not remain in intravascular space but distribute rapidly into the extracellular fluid and the time remaining in intravascular space is much shorter in crystalloids compared with colloids. Therefore the timing of infusion may be the main key to prevent hypotension because the volume expanding effect is maximal at the time of administration.

In this prospective randomized controlled study, we hypothesized that co-load of crystalloid, compared to preload, would be more effective for preserving effective intravascular volume during vasodilatation induced by spinal blockade, and that co-load is useful for preventing maternal hypotension during spinal anesthesia for cesarean delivery.

## Methods

After Institutional Review Board (Seoul National University Bundang Hospital, South Korea) approval and written informed consent, a total of 60 ASA I parturients scheduled for elective cesarean delivery under spinal anesthesia was enrolled. This prospective randomized controlled study followed the CONSORT guidelines. Exclusion criteria were gestational age < 37 wks, multiple gestation, fetal distress, preeclampsia, cardiovascular disease, and diabetes. Study design was registered in Clinical Research Information Service (https://cris.nih.go.kr, KCT0000324).

Venous access was prepared with 18 gauge intravenous catheter on left forearm and the standard monitoring including electrocardiogram, noninvasive blood pressure measurement, and pulse oximetry was applied. Parturients were randomized into one of two groups using computer generated random allocation (block randomization, block size 4). The preload group received rapid infusion of 15 ml/kg of Hartmann’s solution(sodium 131 mmol/L, chloride 111 mmol/L, lactate 29 mmol/L, potassium 5 mmol/L, calcium 2 mmol/L, osmolarity 279 mOsm/L) on arrival in the operating room before spinal anesthesia. The same amount and type of fluid was infused in the coload group, but it was initiated just after intrathecal administration of local anesthetic solution for spinal anesthesia. Before starting spinal anesthesia, systolic blood pressure and heart rate were measured three times in the wedged supine position and the average of 2^nd^ and 3^rd^ value was regarded as baseline.

Spinal anesthesia was conducted in the right lateral decubitus position. After skin infiltration with lidocaine, a 26-gauge spinal needle was inserted at the L 3–4 interspace. After appearance of clear cerebrospinal fluid, 0.5% hyperbaric bupivacaine 8 mg and fentanyl 15 μg were injected. Parturients were then immediately placed in the tilted supine position. Urinary catheter was inserted for all patients. Blood pressure and heart rate were recorded at 1 min interval starting 1 min after intrathecal injection. Pulse oximetry and axillary temperature were monitored continuously. Hypotension as primary outcome, was defined as a decrease of systolic blood pressure by 20% or more from the baseline value and was treated with IV ephedrine in increments of 5 mg. The lowest blood pressure checked was recorded and nausea, vomiting were evaluated until baby birth. Before starting the procedure, parturients were instructed to report if they feel nauseated. The extent of sensory block was checked with pinprick at 3 min interval starting 3 min after intrathecal injection until stabilized. Sedation was not done for any patients. Neonatal Apgar scores were recorded at 1 min and 5 min after delivery and umbilical arterial and venous blood gas analysis was done.

### Statistical Analysis

Sample size was determined by power analysis based on pilot data (desired power = 0.8, alpha = 0.05, hypotension incidence 80% and significant if 50% decrease in incidence) and a minimum of 28 partrients per group was required.

The incidence of hypotension and nausea were evaluated with Chi-square test and ratio-scale data were analyzed and compared by student t-test or rank-sum test if appropriate.

## Results

**Figure 1:**
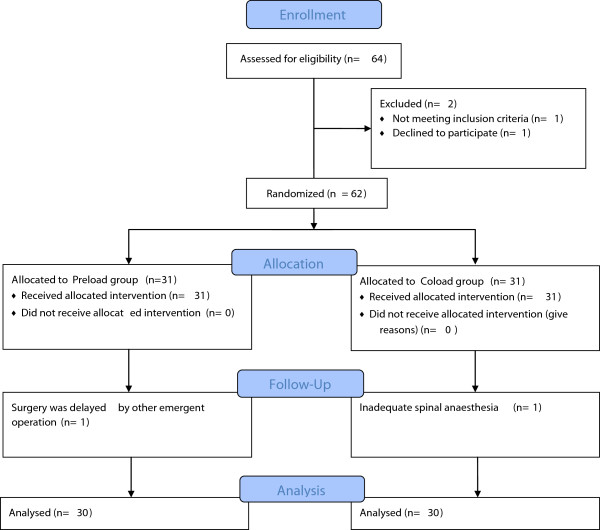
Consort flow diagram.

Finally, a total of 60 parturients completed the study (Figure 1). Parturient characteristics including age, weight, height, and abdominal circumference were comparable as well as the maximal block height after spinal anesthesia (Table 1).

**Table 1:** Demographic data and characteristics of spinal anesthesia

	**Preload group**	**Coload group**
	**(N = 30)**	**(N = 30)**
Age (yr)	33.5 ± 3.5	33.7 ± 4.0
Weight (kg)	72.5 ± 8.0	71.2 ± 7.2
Height (cm)	160.4 ± 3.5	161.2 ± 4.6
Abdominal circumference (cm)	101.3 ± 7.5	101.5 ± 4.6
Maximal block height	T3 [C6-T6]	T3 [C8-T6]
Anesthesia time (min)	86.7 ± 18.0	90.8 ± 19.7

Data are presented as mean ± SD or median [range].

There were no significant differences between both groups.

The incidence of hypotension was significantly lower in the coload group compared to the preload group, that is, more parturients in the preload group needed treatment with ephedrine (83.3 % *vs.* 53.3%, *P* = 0.026). About two-fold amount of ephedrine was administered to parturients of preload group compared to the coload group (15.2 ± 11.9 mg *vs*. 7.5 ± 8.6 mg, *P* = 0.015). The heart rate before anesthesia was lower in preload group (79 ± 10 bpm vs. 86 ± 15 bpm, *P* = 0.035) and the heart rate at the lowest blood pressure was higher in preload group compared to coload group (95 ± 21 bpm vs. 79 ± 14 bpm, *P* = 0.023). The incidence of nausea was also greater in the preload group (60.0% *vs*. 26.7%, *P* = 0.026) (Table 2). No parturient vomited and no other complications such as respiratory failure observed.

**Table 2:** Maternal hypotension and nausea

	**Preload group**	**Coload group**	***P*-value**
	**(N = 30)**	**(N = 30)**	
Systolic blood pressure (SBP, mmHg)			
Pre-operation at ward	113 ± 11	111 ± 11	0.433
Before anesthesia (baseline, a)	116 ± 13	113 ± 8	0.262
Lowest SBP (b)	82 ± 13	88 ± 12	0.093
Delta SBP (a-b)	34 ± 13	25 ± 10	**0.002**
Mean blood pressure (MBP, mmHg)			
Before anesthesia (baseline, c)	78 ± 10	77 ± 9	0.849
Lowest MBP (d)	49 ± 10	57 ± 12	**0.023**
Delta MBP (c-d)	29 ± 11	20 ± 9	**0.011**
Heart rate (bpm)			
Pre-operation at ward	76 ± 10	76 ± 11	0.914
Before anesthesia	79 ± 10	**86 ± 15**	**0.035**
At lowest BP (d)	**95 ± 21**	79 ± 14	**0.023**
Hypotension, N (%)	25/30 (83%)	16/30 (53%)	**0.026**
Ephedrine dose (mg)	15.2 ± 11.9	7.5 ± 8.6	**0.015**
Nausea, N (%)	18/30 (60%)	8/30 (27%)	**0.019**
Vomiting, N (%)	0	0	-

Data are presented as mean ± SD or number (%).

Hypertension was defined lowest SBP lower than 80% of baseline.

Neonatal outcomes, which were measured by Apgar scores and umbilical arterial and venous blood gas analysis, were within normal range and comparable between two groups (Table 3).

**Table 3:** Neonatal outcomes: apgar scores and umbilical venous gas analysis

	**Preload group**	**Coload group**
	**(N = 30)**	**(N = 30)**
Apgar score at 1 min	8 (7–9)	8 (7–9)
Apgar score at 5 min	9 (8–10)	9 (8–10)
Umbilical artery		
pH	7.32 ± 0.06	7.33 ± 0.03
PCO_2_ (mmHg)	48 ± 7	49 ± 6
PO_2_ (mmHg)	22 ± 5	26 ± 6
HCO_3_ (mEq/dl)	25 ± 2	25 ± 2
Umbilical vein		
pH	7.32 ± 0.06	7.35 ± 0.04
PCO_2_ (mmHg)	42 ± 8	44 ± 5
PO_2_ (mmHg)	32 ± 9	31 ± 7
HCO_3_ (mEq/dl)	23 ± 2	25 ± 2

Data are median (range) or mean ± SD.

There were no significant differences between both groups.

## Discussion

This study demonstrated that when administering crystalloids for prevention of maternal hypotension after spinal anesthesia for cesarean delivery, coload is more efficient than preload, that is, administering crystalloids at the actual time of intravascular volume deficit is more efficient than prophylactic administration.

This result is somewhat different from previous ones, majority of which show no superiority of either methods over one [3-5,10]. American Society of Anesthesiologists (ASA) clinical practice guideline recommendation concerning spinal anesthesia for cesarean delivery states: “Although fluid preloading reduces the frequency of maternal hypotension, initiation of spinal anesthesia should not be delayed to administer fixed volume of intravenous fluid [11]”. A recent meta-analysis also concludes that the timing of fluid loading does not have an impact on the incidence of hypotension [10]. However, this analysis combined crystalloids and colloids and only limited data are available for crystalloids. Crystalloids and colloids should be evaluated separately in this respect. It is known that colloids remain in intravascular space longer than crystalloids do. After volume loading in the parturients, 28% of lactated Ringer’s solution and 100% of hydroxyethylstarch solution remained in the vascular space and the percentage increase in blood volume and that of cardiac output had a significant correlation [12]. In this context, it is not surprising that when colloids were administered for prevention of hypotension after spinal anesthesia for cesarean delivery, no significant difference in the incidence of hypotension or vasopressor requirement was found between the preload and coload groups [3-5].

Crystalloid is less effective than colloids in respect of preventing hypotension. This might be due to rapid redistribution of crystalloids on administration and only a small portion of infused fluid is remained in intravascular space at the time of vasodilation after spinal anesthesia [12]. However, there exist only limited data [13] comparing preload and coload of crystalloid for prevention of hypotension after spinal anesthesia for cesarean delivery. They showed that the number of parturients requiring ephedrine and ephedrine dose used at pre-delivery were lower in coload group but the overall ephedrine dose used were not different between the groups and mean arterial pressure were lower in coload group compared to preload group. They concluded that coload of crystalloid may be advantageous rather than preload in terms of maternal blood pressure prior to delivery but some controversy exists.

Our results clearly show that coload of crystalloid is more advantageous than preload because both the incidence of maternal hypotension and the amount of ephedrine used are lower in coload group as well as the incidence of nausea, which seems to be closely related to hypotension. Crystalloids are not confined to intravascular space but rapidly distribute into the extracellular space, so infusing crystalloids at the time of vasodilation are more effective than prophylactic infusion in reducing the hypotension resulting from vasodilation induced by spinal anesthesia. However, the incidence of hypotension in both groups was high (83.3% and 53.3%) and significant number of parturients in group C still needed vasopressor treatment. Recent studies have shown that colloids are more effective than crystalloids for prevention of hypotension after spinal anesthesia for cesarean delivery but the incidence of hypotension remains relatively high regardless of the type of fluids [12,14] and combined use of vasopressor is recommended regardless of the type of fluids administered [2,15]. Additionally, colloids are more expensive and have higher potential risk of allergic reactions. That could be a reason why many practitioners still use crystalloid as a first choice of fluid for prevention of hypotension [16] as well as our institution does.

The limitation of this study is that it was not blinded to an investigator who record blood pressure, though anesthesiologist was blinded when spinal anesthesia was conducted. However, the judgement of hypotension or ephedrine administration was done under clear-cut standard and the effect of this unblinded method on our results might be small. In this study, hypotension was not completely prevented even in the coload group. It should be kept in mind that hydration only is not sufficient for the prevention of maternal hypotension and vasopressor should be always prepared to be administered.

## Conclusions

Hydration with crystalloid is recommended to be done at the time of actual block rather than pre-hydration before the block in parturients undergoing spinal anesthesia for cesarean delivery.

## Competing interests

The authors declare that they have no competing interest.

## Authors’ contributions

AYO contributed to conception and design of the study, wrote the manuscript. JWH contributed to conception and design of the study, revised the manuscript. IAS, MHK, JHR and YTJ contributed to acquisition, analysis, and interpretation of data. HPB and SHD revised the manuscript. All authors read and approved the final manuscript.

## Pre-publication history

The pre-publication history for this paper can be accessed here:

http://www.biomedcentral.com/1471-2253/14/36/prepub
